# Transabdominal ultrasonographic measurement of caudal vena cava to aorta derived ratios in clinically healthy neonatal foals

**DOI:** 10.1002/vms3.506

**Published:** 2021-05-03

**Authors:** Chiara Del Prete, Francesca Freccero, Aliai Lanci, Gayle D. Hallowell, Chiara Bullone, Carolina Castagnetti, Maria Pia Pasolini

**Affiliations:** ^1^ Department of Veterinary Medicine and Animal Production University of Naples Federico II Naples Italy; ^2^ Department of Veterinary Medical Sciences University of Bologna Bologna Italy; ^3^ School of Veterinary Medicine and Science Sutton Bonington Campus University of Nottingham Leicestershire UK; ^4^ Private Practitioner Naples Italy; ^5^ Health Science and Technologies Interdepartmental Center for Industrial Research (HST‐ICIR) University of Bologna Bologna Italy

**Keywords:** caudal vena cava/aorta ratios, foals, intravascular volume, ultrasound

## Abstract

**Background:**

Ultrasonographic measurement of the vena cava and aorta (Ao) diameters and their ratios have been suggested to be a reliable way of quantifying hypovolemia.

**Objective:**

To evaluate the feasibility and reliability of an ultrasonographic technique for measurement of Ao and caudal vena cava (CVC) and derived ratios using three different acoustic windows in a population of healthy neonatal foals. Correlation between Ao and CVC measurements and ratios and foals' age or bodyweight were also investigated.

**Methods:**

In 14 healthy foals aged less than 7 days, the diameters of the Ao and of the CVC in long and short axis were measured by two observers from images obtained using three different ultrasonographic imaging planes (left dorsal, left ventral and right views). The Ao and CVC cross‐sectional area and the CVC/Ao diameter and area ratios were calculated. Image quality was subjectively assessed. Intraobserver and interobserver reliabilities for image quality scores and measurements were evaluated between the two observers. Simple linear regression models were used to identify correlations between the CVC/Ao measurements and ratios and the age and bodyweight of the foals.

**Results:**

The left ventral view showed the highest reliability. A correlation between bodyweight and the short axis measurement of the CVC was found (*R* ^2^ = 0.385; *p* = 0.018). Age was positively correlated with the long axis of measurement of the CVC (*R* ^2^ = 0.426; *p* = 0.011) and CVC/Ao diameter ratio (*R* ^2^ = 0.625; *p* = 0.001).

**Conclusions:**

The left ventral view allows the Ao and CVC cross sections to be easily visualized and measured in neonatal foals in right lateral recumbency.

## Graphical abstract

The present study evaluated the feasibility and reliability of an ultrasonographic technique for measurement of Ao and CVC and derived ratios using three different acoustic windows in a population of healthy neonatal foals. A left ventral view of the abdomen allowed for rapid imaging and measurement of the cross‐sectional diameter of both the aorta and the CVC in neonate foals in lateral recumbency, and might be a promising method to quantitatively assess the degree of hypovolemia.

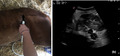

## 1 INTRODUCTION

Hypovolaemia is a common condition encountered in neonatal foals and, if unrecognized and untreated, can become severe and life threatening (Palmer, 2004).

The assessment of the intravascular volume status can be challenging and difficult to achieve.

In human medicine, different non‐invasive methods have been developed to attempt to more accurately assess and quantify the degree of hypovolaemia in both adults and children (Duggan et al., 1996; Gorelick et al., 1997; Saavedra et al., 1991; Steiner & Byerley, 2004; Yilmaz et al., 2002; Zengin et al., 2013; Zöllei et al., 2013). Several studies have demonstrated that the ratios of the inferior vena cava and aorta (Ao) diameters using ultrasonography may be useful non‐invasive parameters to assess hydration status and to predict volume responsiveness (Chen et al., 2007, 2010; Kosiak et al., 2008; Levine et al., 2010; Lyon et al., 2005; Yanagawa et al., 2005). In a recent study by Kwon et al., (2016), it has been proposed that the diagnostic performance of Ao/inferior vena cava cross‐sectional area index for hypovolaemia in children might be superior to using ratios of the diameters of these structures.

Similar studies in veterinary medicine are scarce. Recently, the use of ultrasonographic measurements of the vena caval and aortic diameters and their ratios for the body fluid volume assessment in dogs has been investigated (Cambournac et al., 2018; Darnis et al., 2018; Kwak et al., 2018). Three different transducer placement sites (subxiphoid, hepatic and paralumbar) were used to establish reference intervals for the caudal vena cava (CVC) and Ao diameters, areas and ratios in healthy dogs (Darnis et al., 2018). The ultrasonographic measurements of CVC and Ao, obtained using the spleno‐renal window described by Lisciandro (2011), resulted in reliable and operator‐independent results. Furthermore, ultrasonographic CVC/Ao ratios proved to be a feasible method to quantify volume depletion in beagle dogs (Kwak et al., 2018).

In foals, two studies have described visualization of CVC and Ao in the context of the ultrasonographic assessment of the kidneys and adrenal glands (Aleman et al., 2002; Hoffmann et al., 2000). Recently, Tuplin et al., (2017) investigated the ultrasonographic identification of CVC and CVC collapsibility index at the subxiphoid site in standing healthy foals.

The aims of the present study were (1) to investigate the intraobserver and interobserver reliabilities for image quality and measurements of Ao and CVC diameters obtained using three different ultrasonographic views in healthy neonatal foals; (2) to calculate the CVC/Ao diameter, area and derived ratios and (3) to correlate CVC/Ao measurements and ratios with foals' age and bodyweight. This information is essential before the technique can be used to assess the degree of hypovolaemia in foals.

## 2 MATERIAL AND METHODS

### 2.1 Animals

The study was performed at the ‘blinded for review’. Fourteen healthy newborn foals (nine fillies and five colts) born from mares housed for attended delivery were included in this study: 12 were Standardbreds, and two were Warmbloods. All foals were born at term with APGAR score >8 and had a serum IgG concentration of >800 mg/dl at 18–24 hr of life. Foals were deemed healthy based on physical examination and complete blood count and biochemistry panel at birth. The clinical examination was repeated every 12 hr during the observation period. Foals with any sign of illness or hypovolaemia (tachycardia, weak pulses, poor filling of the jugular vein, tachypnea, cold extremities and prolonged capillary‐refill time) were excluded from the study.

Foals were housed with their dams, turned out in a paddock and free to nurse. All newborn foals hospitalized in the unit are routinely submitted to umbilical region ultrasound once within the first week of life. For this study, ultrasonographically evaluation of the Ao and CVC was performed at the same time of this routinely examination. Consent for the ultrasonographic evaluation was obtained from all owners. Before the ultrasound evaluation, the age (in hours) and bodyweight (in kg), estimated using a weight tape, were recorded. During the examination, foals were not sedated and were manually restrained on a mattress near the mare, in both right and left lateral recumbencies. The hair was not clipped, and alcohol was applied to provide conduction of the ultrasound signal.

### 2.2 Ultrasonography protocol

Two‐dimensional, real‐time ultrasound was performed by one single investigator with diagnostic imaging expertise, using a portable machine (MyLab™Alpha, Esaote, Genova, Italy) equipped with a 1‐ to 8‐MHz convex array transducer set at 6 MHz. Standard ultrasound settings were used in all foals with the depth was set to 10 cm, the focus positioned at the level of Ao and CVC and only the gain was individually adjusted to optimize image quality.

**FIGURE 1:**
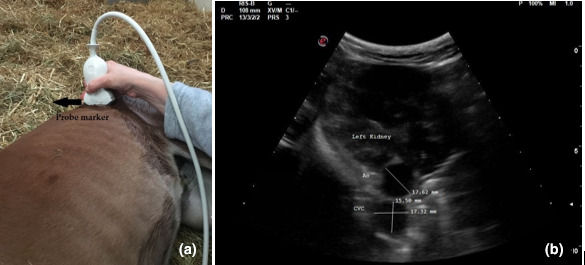
Left dorsal view (View 1): (a) transducer placement site and (b) ultrasonographic measurements of cross‐sectional diameters of the aorta and of the long and short axis of the caudal vena cava

**FIGURE 2:**
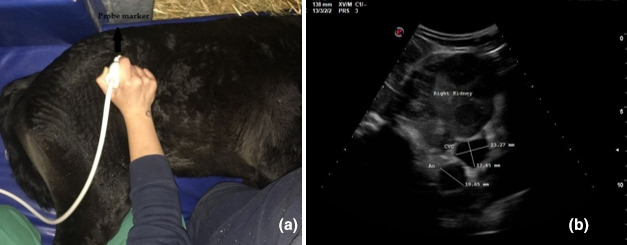
Right view (View 3): (a) transducer placement site and (b) ultrasonographic measurements of cross‐sectional diameters of the aorta and of the long and short axis of the caudal vena cava

**FIGURE 3:**
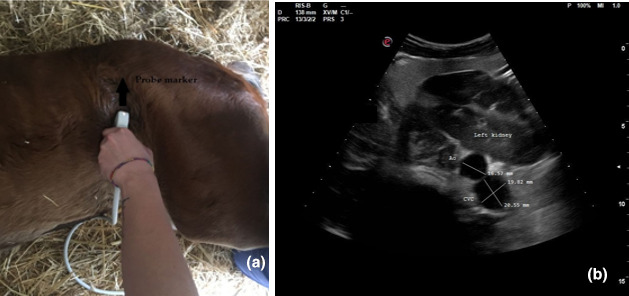
Left ventral view (View 2): (a) transducer placement site and (b) ultrasonographic measurements of cross‐sectional diameters of the aorta and of the long and short axis of the caudal vena cava

In each patient, three ultrasonographic views were evaluated: two from the left side when the foal was in right lateral recumbency and one from the right side when the foal was in left lateral recumbency. On the left and right side, the probe was placed perpendicular to the skin in a transverse position, between the last rib and the tuber coxae, with the probe marker pointing dorsally. Once the left or right kidney was identified, the probe was angled ventrally to visualize the Ao and CVC, deep to the left kidney (left dorsal view, View 1, Figure 1a; right view, View 3, Figure 2a). For the second view on the left (left ventral view, View 2; Figure 3a), the probe was placed beneath the tuber coxae, and the probe angled dorsally in order to identify the left kidney, Ao and CVC.

The Ao was identified by its anatomical position, circular shape, echogenic walls and pulsation observed with each ventricular systole. The CVC was identified by its elliptical shape, and it was visualized deep to the left kidney and the Ao in both the left dorsal and ventral views (Hoffmann et al., 2000). On the right view, the Ao lay deep to the right kidney and CVC appeared deep to the right kidney and ventral to the Ao.

The Ao and CVC were imaged simultaneously in cross section from each ultrasonographic view (Figures 1b, 2b and 3b). One cineloop of at least 10 s was recorded and stored in DICOM format for post hoc analysis.

### 2.3 Ultrasonographic measurements

Following image acquisition in all foals, evaluation of imaging quality and measurements were made by two investigators (observers A and B) with intermediate and basic levels of experience in ultrasound. The investigators performed an independent and blinded review of the cineloops.

The image quality of Ao and CVC was scored using a prefixed 4‐point scale, as shown in Table 1.

**TABLE 1:** Four‐point scale used to score the image quality of the cineloops of Ao and CVC in cross sections obtained using three ultrasonographic imaging planes in healthy neonatal foals

Scores	
0	Poor visualization
1	Both vessels unclear (i.e., gas within the intestinal tract obscuring all or part of the vessels)
2	Margins of ventral vessel unclear
3	Well‐defined visualization of both vessels

Then, two still frames from the cineloops that demonstrated the highest resolution of both Ao and CVC and with the Ao at its largest diameter in ventricular systole were selected for measurement.

The diameter of Ao and measurement of the long and short axis of the CVC elliptical area were made from the cross‐sectional views of the vessels, using the electronic calipers of the ultrasound machine. Each observer repeated each measurement twice, one for each selected frame.

The CVC/Ao diameter index was obtained by dividing the long axis diameter of the CVC by the diameter of the Ao​ (Kosiak et al., 2008). The Ao cross‐sectional area was calculated with the formula of the area of a circle, as [π × (½ of the Ao diameter)^2^] (Kwon et al., 2016). The CVC cross‐sectional area was calculated with the formula of the area of an ellipse, as [π × ½ of the CVC long axis × ½ of the CVC short axis] (Darnis et al., 2018). The CVC/Ao area index was calculated as the ratio between CVC and Ao cross‐sectional areas (Kwak et al., 2018).

### 2.4 Statistical methods

Data were evaluated for normality using the Shapiro–Wilk test. Depending on distribution characteristics, mean ± *SD* (normally distributed data) or median and interquartile range (IQR; non‐normally distributed data) were calculated for all the ultrasound measurements recorded by each observer.

The interobserver reliability for the quality score was performed by comparing the score assigned to each of the three ultrasonographic views by the two investigators. A Cohen's kappa coefficient (*κ*) was calculated (Cohen, 1960). The *κ* values are interpreted as <0 indicating no agreement, 0.20 slight, 0.21–0.40 fair, 0.41–0.60 moderate, 0.61–0.80 substantial and 0.81–1 almost perfect agreement (McHugh, 2012).

Intraobserver reliability for ultrasonographic measurements (Ao diameter, CVC long and short axis diameters) were calculated comparing the two repeated measurements by each observer for the three views, while for the interobserver reliability, the mean of the two measurements by each observer were used for comparison.

Intraclass correlation coefficient (ICC) were also calculated. The ICC estimates and their 95% confidence intervals were calculated based on a mean rating (*k* = 2), absolute agreement, two‐way mixed‐effects model. ICC values were considered to show excellent reliability if they were >0.90, good reliability if between 0.75 and 0.90, moderate for values between 0.50 and 0.75 and poor reliability for values < 0.50 (Koo & Li, 2016).

On the view with best results of reliability were conducted further analysis. Intraobserver and interobserver variabilities in ultrasound measurements of Ao diameter and short and long axis of CVC measurements were calculated as the absolute difference between the two measurements divided by the mean value of observers' measurements and expressed as a percentage.

Simple linear regression models were used to identify correlations between the ultrasound measurements or the indices and the age (in hours) or the estimated bodyweight (kg) of the foal. For each regression, a coefficient of determination (*R* ^2^), regression coefficient (*β*) and *p* value were estimated. Significance level was set at *p* < 0.05. Statistical analysis was performed using SPSS version 25.0 (SPSS Inc., Chicago, IL, USA).

## 3 RESULTS

Foals included in this study were aged 12 to 164 hr (mean 59.9 ± 52.1 hr). Bodyweight ranged between 41 and 71 kg (median 52 kg; IQR 46 and 56.5 kg).

Cineloops of diagnostic quality were acquired from the three different ultrasound views in all 14 patients. The time needed to find the optimal window to visualize simultaneously Ao and CVC in their cross section was not recorded. Image evaluation and measurements were deemed easy by both investigators.

**FIGURE 4:**
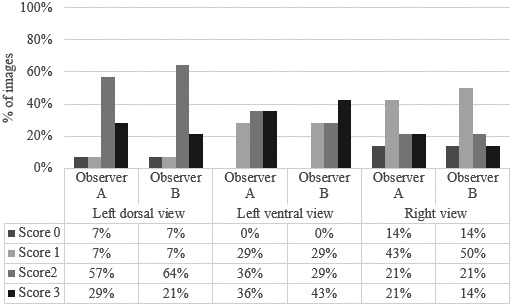
Graphic representation and table of the percentage of images of Ao and CVC assigned to each quality score for the left dorsal, left ventral and right ultrasonographic views. The imaging quality of cineloops was scored on a four‐point scale (0 = poor visualization, 1 = both vessels unclear, 2 = ventral vessel unclear and 3 = well‐defined visualization of both vessels) by observers A and B independently

The percentage of the image quality scores assigned by each observer for the right, left dorsal and left ventral views are displayed in Figure 4. The quality score was deemed satisfactory for all views. The highest scores were reported for the left ventral view (highest percentage score of 3; Figure 4), and this view was never assigned a score of 0 (0%), unlike the other two views. The limitation of the right view was the gas content into the intestine impacting on vessel visualization.

The interobserver reliability of the quality score as Cohen's *κ* values are reported in Table 2. No (*κ* = 0.109), fair (*κ* = 0.274) and moderate agreement (*κ* = 0.569) were observed for the left dorsal, right and left ventral views, respectively. Table 2 further shows the ICCs for intraobserver and interobserver reliability calculated for all the ultrasound measurements. Based on the previously published cut‐offs (Koo & Li, 2016), the ICC for interobserver reliability of the Ao diameter was poor (0.32) in the right view, whereas the interobserver ICC of CVC short axis was excellent in this view (0.90). The ICC for intraobserver reliability for the right view was deemed to be ‘moderate’ to ‘excellent’ (0.70 to 0.93). In the left dorsal view, the intraobserver reliability for all measurements were good to excellent for both observers, ranging from 0.78 to 0.95; the interobserver ICC was moderate for the long axis of CVC (0.59). As shown in Table 2, both the intraobserver and interobserver ICCs in the left ventral view were good to excellent (ICCs > 0.85).

**TABLE 2:** Interobserver reliability for imaging quality score and intraobserver and interobserver reliabilities for ultrasonographic measurements of Ao and CVC obtained using three ultrasonographic views (left dorsal, left ventral and right) in healthy neonatal foals

*n* = 14	Left dorsal view				Left ventral view				Right view			
*κ* values and ICCs (95% CI)	Quality score	Ao diameter	CVC long axis	CVC short axis	Quality score	Ao diameter	CVC long axis	CVC short axis	Quality Score	Ao diameter	CVC long axis	CVC short axis
Intraobserver reliability (Observer A)	/	0.78 (0.31–0.92)	0.90 (0.68–0.97)	0.90 (0.71–9.70)	/	0.91 (7.55–9.73)	0.87 (0.65–0.96)	0.98 (0.94–0.99)	/	0.92 (0.75–0.97)	0.70 (0.27–0.91)	0.88 (0.63–0.96)
Intraobserver reliability (Observer B)	/	0.95 (0.85–0.98)	0.84 (0.51–0.95)	0.90 (0.71–0.97)	/	0.91 (0.74–0.97)	0.93 (0.78–0.98)	0.91 (0.72–0.97)	/	0.76 (0.23–0.93)	0.93 (0.80–0.98)	0.92 (0.76–0.98)
Interobserver reliability (A vs. B)	1.09 (−0.36 to 0.58)	0.81 (0.40 to 0.94)	0.59 (−0.34 to 0.87)	0.79 (0.33–0.93)	0.57 (0.21–0.92)	0.91 (0.73–0.97)	0.85 (0.54–0.95)	0.89 (0.67–0.96)	0.27 (−0.93 to 0.64)	0.32 (−1.37 to 0.79)	0.71 (0.09–0.91)	0.90 (0.70–0.97)

Note

*κ* and ICC values: <0 no agreement, 0.20 slight agreement, 0.21–0.40 fair agreement, 0.41–0.60 moderate agreement, 0.61–0.80 substantial agreement and 0.81–1.0 almost perfect agreement. ICC values: <0.5 poor reliability, 0.5–0.75 moderate reliability, 0.75–0.9 good reliability and >0.90 excellent reliability.

Abbreviations: Ao, aorta; CVC, caudal vena cava; ICC, intraclass correlation coefficient.

Based on the previous results, the left ventral view was deemed the best performing view and was used for further analysis. Intraobserver variability of Ao diameter and short and long axis of CVC in the left ventral view were between 7.4% and 10.8% for observer A and 5.9% and 13% for observer B (Table 2). The highest intraobserver variability was found in the CVC long axis for observer A (10.8%) and in the CVC short axis for the observer B (13%). Interobserver variability was lowest for the Ao diameter (6.6%), 13.6% for CVC short axis and the highest for the CVC long axis (14.5%; Table 2).

All ultrasound measurements (Ao diameter, long and short axis of CVC) and the calculated values (CVC/Ao diameter index, Ao area, CVC area, CVC/Ao area index) from the left ventral view are reported as mean (±*SD*) or median (IQR) in Table 3.

**TABLE 3:** Ultrasonographic measurements and calculated variables obtained on images from the left ventral view (View 2) by two observers in healthy neonatal foals

Mean ± *SD*/median (IQR)	Ao diameter (mm)	CVC long axis (mm)	CVC short axis (mm)	CVC/Ao diameter index	Ao area (mm^2^)	CVC area (mm^2^)	CVC/Ao area index
Observer A	16.0 ± 2.1	16.5 ± 2.5	14.1 ± 4	10.5 ± 1.9	20.7 ± 5.4	16.4 (13.8–19.8)	9.1 ± 2.8
Observer B	16.4 ± 1.6	17.1 ± 3.7	14.9 ± 3.0	10.6 ± 2.5	21.2 ± 4.2	18.8 (16.1–21.4)	9.7 ± 3.6
A–B mean	16.2 ± 1.8	16.8 ± 2.9	14.5 ± 3.4	10.5 ± 2.1	21 ± 4.6	18.0 (14.5–21)	9.4 ± 3

Note

Measured variables: Ao diameter, CVC long axis diameter and CVC short axis diameter. Calculated variables: CVC/Ao diameter index: CVC long axis/Ao diameter; Ao area: 3.14 × (½ of the Ao diameter)^2^; CVC area: 3.14 × ½ CVC long axis × ½ CVC short axis; CVC/Ao area index: CVC area/ Ao area. *N* = 14 foals.

**FIGURE 5:**
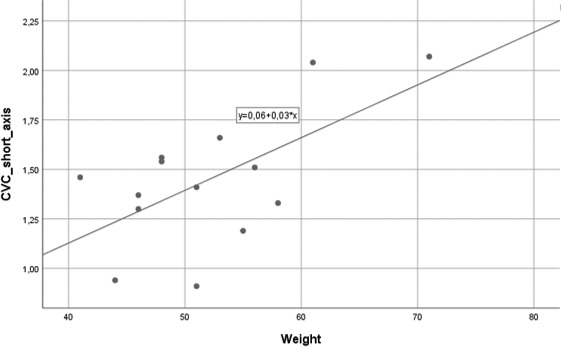
Linear correlation between foals' body weight (kg) and the short axis diameter of CVC (mm) (*R* ^2^ = 0.385; *β* = 0.620; *p* = 0.018)

**FIGURE 6:**
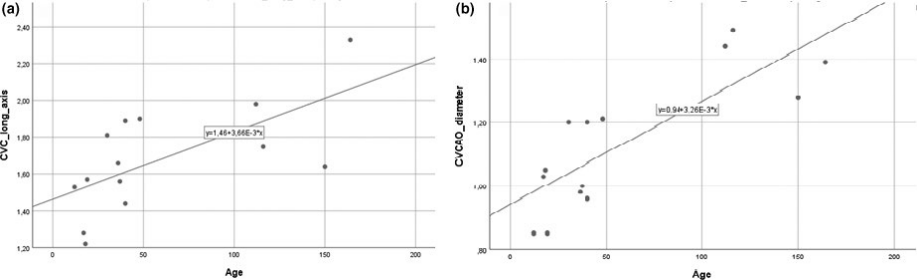
(a) Linear correlation between foals' age (h) and CVC long axis diameter (mm) (*R* ^2^ = 0.426; *β* = 0.653; *p* = 0.011) and (b) the CVC/Ao diameter index (*R* ^2^ = 0.625; *β* = 0.808; *p* = 0.001)

A positive linear correlation between estimated bodyweight and the short axis of CVC (*R* ^2^ = 0.385; *β* = 0.620; *p* = 0.018) was found (Figure 5). Age was also positively correlated with CVC long axis (*R* ^2^ = 0.426; *β* = 0.653; *p* = 0.011; Figure 6a) and CVC/Ao diameter index (*R* ^2^ = 0.625; *β* = 0.808; *p* = 0.001; Figure 6b). No other correlations were identified.

## 4 DISCUSSION

In this study, a probe placement site that allowed for rapid imaging and measurement of the cross‐sectional diameter of both the Ao and the CVC in newborn foals was identified in a left ventral view of the abdomen. This proved to be a non‐invasive procedure that was well tolerated in healthy foals and might be a promising method to quantitatively assess the degree of hypovolaemia either in a hospital setting or in the field. This was the first step to determine if such an ultrasound technique was viable in foals.

In this study, the position of the patient was dictated by the fact that most of the sick foals are examined in lateral recumbency. Moreover, in our experience, foals in lateral recumbency are more relaxed during ultrasound examination. In human medicine, it has been shown that the position of the patient influences CVC diameter, which is smallest in left lateral recumbency, largest in right lateral recumbency, with dorsal recumbency providing intermediate values (Ciozda et al., 2015). These findings in humans may not be relevant to veterinary species due to differences in thoracic shape. In order to ultrasonographically visualize CVC in dogs, the patients were positioned in left (Darnis et al., 2018; Kwak et al., 2018; Meneghini et al., 2016) and right (Cambournac et al., 2018) lateral recumbency also and found both sites reliable in measuring CVC diameter. Only the standing position was chosen to measure the CVC diameter and collapsibility index in foals in one study (Tuplin et al., 2017). In our study, the influence of foal position on the measurements or indices of Ao and CVC were not evaluated, and thus, this aspect needs further investigation.

As previously described, both right and left kidneys, Ao and CVC could be imaged by placing the probe in the ipsilateral paralumbar fossa or 17th intercostal space in neonatal foals (Hoffmann et al., 2000). In this study, the transducer was placed in the paralumbar fossa between the last rib and the tuber coxae, as described in dogs (Cambournac et al., 2018; Darnis et al., 2018). Specifically, the left ventral view was similar to the right paralumbar view used by Darnis et al., (2018) to obtain ultrasonographic reference values of the CVC in healthy adult dogs. The left dorsal view was analogous to the scanning window employed by Cambournac et al., (2018) in the ultrasonographic assessment of volume status using CVC, Ao and CVC/Ao ratios in healthy dogs before and after blood donation. In this study, although the Ao and the CVC could be easily and simultaneously imaged from the three different imaging windows, they were best visualized from the left side when the foal was in right lateral recumbency. In this position, the Ao and CVC appear medial to the ventral aspect of the left kidney, confirming the results obtained by Hoffmann et al., (2000). On the right side, the presence of gas within the cecum can produce a strongly echogenic region interfering with visualization of the vessels. On the left side, the ventral view provides a clearer image of both vessels, as the spleen acts as acoustic window (Boussuges et al., 2009) and the thickness of abdomen wall is thinner. Moreover, in both dorsal views, gas in the intestinal lumen is directed towards the probe causing reverberation artefacts.

Similar to human studies, the transverse plane was used to image the vessels (Chen et al., 2007, 2010; Kathuria et al., 2015; Kwon et al., 2016). Kwak et al.^,^ (2018) also recommended that the transverse plane should be used to measure CVC diameters in dogs. The cylindrical shape of the vessel and mediolateral movements during breathing relative to the transducer could prevent measuring the maximal diameter of the CVC consistently in the longitudinal plane.

According to our results, the left ventral scanning window was overall the easiest and the most reliable view for simultaneous visualization and measurement of Ao and CVC in cross section.

The overall interobserver agreement rates suggested that in clinical practice, different observers could reliably perform the measurements on the same animal at different time points. A degree of intraobserver and interobserver variabilities may be partly attributed to differences between the frames selected. These can reflect true differences in the cross‐sectional diameters due to multiple factors, that is, changes in patient or probe position, change in pressure, different image quality, that is, movement of intraluminal gas or error in the measurement. Moreover, the difficulty in obtaining precise measurements using calipers could be another possible reason of the variability between measurements (Long et al., 2012).

The largest variation for both intraobserver and interobserver measurements were found for CVC in both long and short axis, while the measurements of the Ao diameter showed the lowest variability. This may be a result that veins, unlike arteries, can be easily compressed by the pressure applied by the probe, which would falsely decrease the size of the CVC. However, in dorsal views, the kidney on the left is just behind or under the last rib, next to the spinal cord, and these structures may limit transducer pressure applied to the vessels (Cambournac et al., 2018). It is also known that CVC diameter is larger during expiration than inspiration in foals (Tuplin et al., 2017), and this could be another cause for the variability of these measurements. The American Society of Echocardiography does not specify an optimal phase of the respiratory cycle to measure the maximal CVC diameter (Ciozda et al., 2015). In this study, a transabdominal window was used, which should have negated much of this variation, as the CVC diameter does not tend to change with the respiratory cycle in this location as much (Darnis et al., 2019). Indeed, it was proven in dogs that respiratory movements influenced CVC values minimally at the paralumbar sites when compared with the hepatic view (Darnis et al., 2018).

It has been hypothesized that only measuring the maximum long axis diameter of CVC might not reflect the fluid status of the patient, and that the CVC area might be more sensitive (Kwon et al., 2016). In addition, the use of indices, that is, CVC/Ao diameters or cross‐sectional areas ratios, would represent an easy method to normalize the values and to decrease the high variability present in solely measuring the diameter of the CVC (Chen et al., 2007; Kwak et al., 2018). The results obtained in the present study cannot be compared with the literature, because no previous studies had been carried out on ultrasound measurement and indices of Ao and CVC in foals. Therefore, the ranges obtained in our study can be used as benchmarks for future studies.

In the present study, weight and age were positively correlated with CVC measurements in short axis and long axis, respectively. The best correlation was achieved with age and CVC/Ao diameter index (62.5% of the variation in CVC/Ao diameter can be attributed to age). The CVC diameter of normovolaemic children was also found to be positively correlated with age (Kathuria et al., 2015). Furthermore, the aortic diameter of children has been correlated with age, body surface area, height and weight (Poutanen et al., 2003). Darnis et al., (2018) support the use of allometric scaling to predict CVC and Ao measurements according to the dog's weight. No studies currently describe changes of Ao or CVC diameter in foals regarding age or weight. Based on this study, age and bodyweight need to be considered when being used clinically, although this would be of less importance clinically when an animal would be being compared with itself over time. Further studies with a larger sample size and greater representation of breeds is required to confirm this relationship and to determine ranges in different age or weight groups.

In a clinical setting, there are time, financial and ethical limitations in performing repeated ultrasound examinations on the same patient if it is not part of a diagnostic evaluation. Moreover, it is well known that ultrasound is an operator‐dependent technique. In future studies, a less‐experienced ultrasonographer could be enrolled to investigate the influence of the technical skills on the application of the technique. However, in both human and veterinary medicine, ultrasonographic measurement of the CVC and Ao were reliable and considered a simple technique after a brief training session (Darnis et al., 2019; Gustafsson et al., 2015).

Despite several limitations, these results demonstrated that the left ventral view offers the highest reliability for the measurement of the Ao diameter and CVC measurements in short and long axis. Further work is warranted to investigate if the same ultrasound view can be applied to older foals. The larger size and the presence of a more developed large intestine in older subjects (Aleman et al., 2002) may reduce success in visualizing the entire Ao and CVC.

In the present study, only healthy foals were enrolled. The clinical applicability and performance of either the diameter or area indices in detecting reductions in intravascular volume and changes following fluid resuscitation need to be evaluated in sick foals. These critically ill patients may have more intraluminal gas or other diseases of the abdomen that could impact on the imaging quality and measurements of the Ao and the CVC.

## 5 CONCLUSION

In conclusion, in the authors' opinion, the calculation of the CVC/Ao ratios obtained by means of the left ventral ultrasonographic window warrants further investigations as potential tool to assess and monitor intravascular volume status in newborn foals.

## CONFLICT OF INTEREST

The authors declare no conflict of interest.

## AUTHOR CONTRIBUTION

**Chiara Del Prete:** Data curation; Methodology; Resources; Writing‐original draft; Writing‐review & editing. **Francesca Freccero:** Methodology; Resources; Supervision; Visualization; Writing‐review & editing. **Aliai Lanci:** Resources; Writing‐review & editing. **Gayle Hallowell:** Supervision; Writing‐review & editing. **Chiara Bullone:** Resources; Writing‐original draft. **Carolina Castagnetti:** Supervision; Writing‐review & editing. **Maria Pia Pasolini:** Conceptualization; Methodology; Supervision; Writing‐review & editing.

## ETHICS STATEMENT

The study has been approved by the ethics review committee at the University of Bologna. An informed client consent has been given for each animal.

### PEER REVIEW

The peer review history for this article is available at https://publons.com/publon/10.1002/vms3.506.

## References

[vms3506-bib-0001] Aleman, M., Gillis, C. L., Nieto, J. E., Renaudin, C. D., & Bea, J. (2002). Ultrasonographic anatomy and biometric analysis of the thoracic and abdominal organs in healthy foals from birth to age 6 months. Equine Veterinary Journal, 34(7), 649–655. 12455834 10.2746/042516402776250414

[vms3506-bib-0002] Boussuges, A., Gole, Y., & Blanc, P. (2009). Diaphragmatic motion studied by m‐mode ultrasonography: Methods, reproducibility, and normal values. Chest, 135(2), 391–400. 10.1378/chest.08-1541 19017880

[vms3506-bib-0003] Cambournac, M., Goy‐Thollot, I., Violé, A., Boisvineau, C., Pouzot‐Nevoret, C., & Barthélemy, A. (2018). Sonographic assessment of volaemia: Development and validation of a new method in dogs. Journal of Small Animal Practice, 59(3), 174–182. 10.1111/jsap.12759 28960319

[vms3506-bib-0004] Chen, L., Hsiao, A., Langhan, M., Riera, A., & Santucci, K. A. (2010). Use of bedside ultrasound to assess degree of dehydration in children with gastroenteritis. Academic Emergency Medicine, 17(10), 1042–1047. 10.1111/j.1553-2712.2010.00873.x 21040104 PMC3058669

[vms3506-bib-0005] Chen, L., Kim, Y., & Santucci, K. A. (2007). Use of ultrasound measurement of the inferior vena cava diameter as an objective tool in the assessment of children with clinical dehydration. Academic Emergency Medicine, 14(10), 841–845. 10.1197/j.aem.2007.06.040 17898246

[vms3506-bib-0006] Ciozda, W., Kedan, I., Kehl, D. W., Zimmer, R., Khandwalla, R., & Kimchi, A. (2015). The efficacy of sonographic measurement of inferior vena cava diameter as an estimate of central venous pressure. Cardiovascular Ultrasound, 14(1), 33. 10.1186/s12947-016-0076-1 PMC4992235 27542597

[vms3506-bib-0007] Cohen, J. (1960). A coefficient of agreement for nominal scales. Educational and Psychological Measurement, 20(1), 37–46. 10.1177/001316446002000104

[vms3506-bib-0008] Darnis, E., Boysen, S., Merveille, A. C., Desquilbet, L., Chalhoub, S., & Gommeren, K. (2018). Establishment of reference values of the caudal vena cava by fast‐ultrasonography through different views in healthy dogs. Journal of Veterinary Internal Medicine, 32(4), 1308–1318. 10.1111/jvim.15136 29749656 PMC6060313

[vms3506-bib-0009] Darnis, E., Merveille, A. C., Desquilbet, L., Boysen, S., & Gommeren, K. (2019). Interobserver agreement between non‐cardiologist veterinarians and a cardiologist after a 6‐hour training course for echographic evaluation of basic echocardiographic parameters and caudal vena cava diameter in 15 healthy Beagles. Journal of Veterinary Emergency and Critical Care, 29(5), 495–504. 31453666 10.1111/vec.12883

[vms3506-bib-0010] Duggan, C., Refat, M., Hashem, M., Wolff, M., Fayad, I., & Santosham, M. (1996). How valid are clinical signs of dehydration in infants? Journal of Pediatric Gastroenterology and Nutrition, 22(1), 56–61. 10.1097/00005176-199601000-00009 8788288

[vms3506-bib-0011] Gorelick, M. H., Shaw, K. N., & Murphy, K. O. (1997). Validity and reliability of clinical signs in the diagnosis of dehydration in children. Pediatrics, 99(5), e6. 10.1542/peds.99.5.e6 9113963

[vms3506-bib-0012] Gustafsson, M., Alehagen, U., & Johansson, P. (2015). Pocket‐sized ultrasound examination of fluid imbalance in patients with heart failure: A pilot and feasibility study of heart failure nurses without prior experience of ultrasonography. European Journal of Cardiovascular Nursing, 14(4), 294–302. 10.1177/1474515114559435 25376773

[vms3506-bib-0013] Hoffmann, K. L., Wood, A. K. W., & McCarthy, P. H. (2000). Ultrasonography of the equine neonatal kidney. Equine Veterinary Journal, 32(2), 109–113. 10.2746/042516400777591615 10743965

[vms3506-bib-0014] Kathuria, N., Ng, L., Saul, T., & Lewiss, R. E. (2015). The baseline diameter of the inferior vena cava measured by sonography increases with age in normovolemic children. Journal of Ultrasound in Medicine, 34(6), 1091–1096. 10.7863/ultra.34.6.1091 26014329

[vms3506-bib-0015] Koo, T. K., & Li, M. Y. (2016). A guideline of selecting and reporting intraclass correlation coefficients for reliability research. Journal of Chiropractic Medicine, 15(2), 155–163. 10.1016/j.jcm.2016.02.012 27330520 PMC4913118

[vms3506-bib-0016] Kosiak, W., Swieton, D., & Piskunowicz, M. (2008). Sonographic inferior vena cava/aorta diameter index, a new approach to the body fluid status assessment in children and young adults in emergency ultrasound—preliminary study. The American Journal of Emergency Medicine, 26(3), 320–325. 10.1016/j.ajem.2007.07.012 18358944

[vms3506-bib-0017] Kwak, J., Yoon, H., Kim, J., Kim, M., & Eom, K. (2018). Ultrasonographic measurement of caudal vena cava to aorta ratios for determination of volume depletion in normal beagle dogs. Veterinary Radiology & Ultrasound, 59(2), 203–211. 10.1111/vru.12566 29024163

[vms3506-bib-0018] Kwon, H., Jung, J. Y., Lee, J. H., Kwak, Y. H., Kim, D. K., Jung, J. H., Chang, I. W., & Kim, K. (2016). Sonographic aorta/IVC cross‐sectional area index for evaluation of dehydration in children. The American Journal of Emergency Medicine, 34(9), 1840–1844. 10.1016/j.ajem.2016.06.060 27339224

[vms3506-bib-0019] Levine, A. C., Shah, S. P., Umulisa, I., Mark Munyaneza, R. B., Dushimiyimana, J. M., Stegmann, K., Musavuli, J., Ngabitsinze, P., Stulac, S., Epino, H. M., & Noble, V. E. (2010). Ultrasound assessment of severe dehydration in children with diarrhea and vomiting. Academic Emergency Medicine, 17(10), 1035–1041. 10.1111/j.1553-2712.2010.00830.x 21040103

[vms3506-bib-0020] Lisciandro, G. R. (2011). Abdominal and thoracic focused assessment with sonography for trauma, triage, and monitoring in small animals. Journal of Veterinary Emergency and Critical Care, 21(2), 104–122. 10.1111/j.1476-4431.2011.00626.x 21463438

[vms3506-bib-0021] Long, A., Rouet, L., Lindholt, J. S., & Allaire, E. (2012). Measuring the maximum diameter of native abdominal aortic aneurysms: Review and critical analysis. European Journal of Vascular and Endovascular Surgery, 43(5), 515–524. 10.1016/j.ejvs.2012.01.018 22336051

[vms3506-bib-0022] Lyon, M., Blaivas, M., & Brannam, L. (2005). Sonographic measurement of the inferior vena cava as a marker of blood loss. The American Journal of Emergency Medicine, 23(1), 45–50. 10.1016/j.ajem.2004.01.004 15672337

[vms3506-bib-0023] McHugh, M. L. (2012). Interrater reliability: The kappa statistic. Biochemia Medica: Biochemia Medica, 22(3), 276–282. 10.11613/BM.2012.031 23092060 PMC3900052

[vms3506-bib-0024] Meneghini, C., Rabozzi, R., & Franci, P. (2016). Correlation of the ratio of caudal vena cava diameter and aorta diameter with systolic pressure variation in anesthetized dogs. American Journal of Veterinary Research, 77(2), 137–143. 10.2460/ajvr.77.2.137 27027706

[vms3506-bib-0025] Palmer, J. E. (2004). Fluid therapy in the neonate: Not your mother's fluid space. The Veterinary clinics of North America: Equine Practice, 20(1), 63–75. 10.1016/j.cveq.2003.11.005 15062459

[vms3506-bib-0026] Poutanen, T., Tikanoja, T., Sairanen, H., & Jokinen, E. (2003). Normal aortic dimensions and flow in 168 children and young adults. Clinical Physiology and Functional Imaging, 23(4), 224–229. 10.1046/j.1475-097X.2003.00501.x 12914562

[vms3506-bib-0027] Saavedra, J. M., Harris, G. D., Li, S., & Finberg, L. (1991). Capillary refilling (skin turgor) in the assessment of dehydration. American Journal of Diseases of Children, 145(3), 296–298. 2003478 10.1001/archpedi.1991.02160030064022

[vms3506-bib-0028] Steiner, M. J., DeWalt, D. A., & Byerley, J. S. (2004). Is this child dehydrated? JAMA, 291(22), 2746–2754. 10.1001/jama.291.22.2746 15187057

[vms3506-bib-0029] Tuplin, M. C., Romero, A. E., & Boysen, S. R. (2017). Influence of the respiratory cycle on caudal vena cava diameter measured by sonography in healthy foals: A pilot study. Journal of Veterinary Internal Medicine, 31(5), 1556–1562. 10.1111/jvim.14793 28766820 PMC5598903

[vms3506-bib-0030] Yanagawa, Y., Nishi, K., Sakamoto, T., & Okada, Y. (2005). Early diagnosis of hypovolemic shock by sonographic measurement of inferior vena cava in trauma patients. Journal of Trauma and Acute Care Surgery, 58(4), 825–829. 10.1097/01.TA.0000145085.42116.A7 15824662

[vms3506-bib-0031] Yilmaz, K., Karaböcüoglu, M., Citak, A., & Uzel, N. (2002). Evaluation of laboratory tests in dehydrated children with acute gastroenteritis. Journal of Paediatrics and Child Health, 38(3), 226–228. 10.1046/j.1440-1754.2002.00792.x 12047687

[vms3506-bib-0032] Zengin, S., Al, B., Genc, S., Yildirim, C., Ercan, S., Dogan, M., & Altunbas, G. (2013). Role of inferior vena cava and right ventricular diameter in assessment of volume status: A comparative study: Ultrasound and hypovolemia. The American Journal of Emergency Medicine, 31(5), 763–767. 10.1016/j.ajem.2012.10.013 23602752

[vms3506-bib-0033] Zöllei, É., Bertalan, V., Németh, A., Csábi, P., László, I., Kaszaki, J., & Rudas, L. (2013). Non‐invasive detection of hypovolemia or fluid responsiveness in spontaneously breathing subjects. BMC Anesthesiology, 13(1), 40. 10.1186/1471-2253-13-40 24188480 PMC3829671

